# Bioinformatic Analysis of miR-200b/429 and Hub Gene Network in Cervical Cancer

**DOI:** 10.1007/s10528-023-10356-2

**Published:** 2023-03-07

**Authors:** Vaibhav Shukla, Sandeep Mallya, Divya Adiga, S. Sriharikrishnaa, Sanjiban Chakrabarty, Shama Prasada Kabekkodu

**Affiliations:** 1 Department of Cell and Molecular Biology, Manipal School of Life Sciences, Manipal Academy of Higher Education, Manipal, India; 2 Department of Bioinformatics, Manipal School of Life Sciences, Manipal Academy of Higher Education, Manipal, India; 3 Center for DNA Repair and Genome Stability (CDRGS), Manipal Academy of Higher Education, Manipal, Karnataka India

**Keywords:** Cervical cancer, miR-200b/429 cluster, Prognosis, Bioinformatic analysis, TCGA

## Abstract

The miR-200b/429 located at 1p36 is a highly conserved miRNA cluster emerging as a critical regulator of cervical cancer. Using publicly available miRNA expression data from TCGA and GEO followed by independent validation, we aimed to identify the association between miR-200b/429 expression and cervical cancer. miR-200b/429 cluster was significantly overexpressed in cancer samples compared to normal samples. miR-200b/429 expression did not correlate with patient survival; however, its overexpression correlated with histological type. Protein–protein interaction analysis of 90 target genes of miR-200b/429 identified EZH2, FLT1, IGF2, IRS1, JUN, KDR, SOX2, MYB, ZEB1, and TIMP2 as the top ten hub genes. PI3K–AKT and MAPK signaling pathways emerged as major target pathways of miR-200b/429 and their hub genes. Kaplan–Meier survival analysis showed the expression of seven miR-200b/429 target genes (EZH2, FLT1, IGF2, IRS1, JUN, SOX2, and TIMP2) to influence the overall survival of patients. The miR-200a-3p and miR-200b-5p could help predict cervical cancer with metastatic potential. The cancer hallmark enrichment analysis showed hub genes to promote growth, sustained proliferation, resistance to apoptosis, induction of angiogenesis, activation of invasion, and metastasis, enabling replicative immortality, evading immune destruction, and tumor-promoting inflammation. The drug–gene interaction analysis identified 182 potential drugs to interact with 27 target genes of miR-200b/429 with paclitaxel, doxorubicin, dabrafenib, bortezomib, docetaxel, ABT-199, eribulin, vorinostat, etoposide, and mitoxantrone emerging as the top ten best candidate drugs. Taken together, miR-200b/429 and associated hub genes can be helpful for prognostic application and clinical management of cervical cancer.

**Supplementary Information:**

The online version contains supplementary material available at 10.1007/s10528-023-10356-2.

## Introduction

Cervical cancer is a malignancy of the cervix, with 570 000 cases and 311 000 deaths globally in 2018 (Arbyn et al. 2020). High-risk human papillomavirus (HPV) infection and genetic and epigenetic changes are strongly linked to cervical cancer development (Mac and Moody 2020). It is the second most common cancer among women aged between 15 and 44, with an estimated age-standardized incidence of 13.1/100000 worldwide. Over one-third of the global cervical cancer load is contributed together by India and China (Arbyn et al. 2020). The relative 5-year survival rate for stage I, stage II, stage III, and stage IV cervical cancer were 83.5%, 80.6%, 66.0%, and 37.1%, respectively (Balasubramaniam et al. 2020). Early detection and understanding of the molecular complexity are proposed to reduce the high mortality rate of advanced stage and distant cervical cancer (Mishra et al. 2011). Despite the availability of sensitive and specific screening tools for early detection, many cervical cancer cases are detected at an advanced stage, contributing to high mortality and morbidity. Hence, there is a need to (i) identify the marker for early diagnosis, prognosis, and therapeutic applications and (ii) a molecular mechanism for better management of cervical cancer. Towards this, analyzing the epigenome may provide sensitive and specific markers for the clinical management of cervical cancer.

Epi-genome-wide studies using microarray and next-generation sequencing have identified the global epi(genomic) changes responsible for carcinogenesis and provided an opportunity to translate these molecular changes as markers for improved clinical management of cervical cancer (Varghese et al. 2018). Nevertheless, the findings from the genome-wide study required cross-validation as the markers identified may be inconsistent because of (i) tumor heterogeneity, (ii) techniques used, (iii) sample types and source, and (iv) algorithms used for data analysis (Hamid et al. 2009). Hence, reanalysis of big data may provide new insights into the regulatory factors, molecular mechanisms, and signaling pathways altered during cervical cancer. Resistance to treatment and lack of effective therapy contributes to a high mortality rate in the advanced stage of cervical cancer (Mehta et al. 2017). Previous in silico analyses have suggested that the reanalysis of big data can identify reliable diagnostic and prognostic markers for the clinical management of cervical cancer (Shukla et al. 2021).

The miR-200b/429 located at 1p36 is a conserved miRNA cluster encoding for three miRNAs, namely miR-200b, miR-200a, and miR-429. Abnormal expression of members of miR-200b/429 have been reported in cervical cancer tissue and cell lines (González-Quintana et al. 2016; Zeng et al. 2016; Fan et al. 2017). Several malignancies have shown dysregulated expression of the miR-200b/429 family (Nam et al. 2008; Mateescu et al. 2011). miR-200b/429 cluster has been demonstrated to target the distinct genes at different phases of cancer growth, even in the same tumor. When miR-200b/429 cluster targets zinc finger E-box-binding homeobox1/2 (ZEB1/2), they prevent local invasion in breast cancer, but when they target SEC23a, they encourage metastatic lung colonization (Korpal et al. 2011). The small RNA sequencing of normal and cervical cancer patients showed miR-200b/429 as one of the most differentially expressed miRNA clusters as per our study (Shukla et al. 2019). It is a potential biomarker for locally advanced radioresistant cervical cancer (Nilsen et al. 2022). Besides, its expression was dysregulated in the The Cancer Genome Atlas-Cervical Squamous Cell Carcinoma (TCGA-CESC) datasets. Functional studies have suggested that the members of this cluster regulated proliferation, migration, and invasion and showed both tumor-suppressive and oncogenic functions in cervical cancer (Hu et al. 2010; González-Quintana et al. 2016; Zeng et al. 2016; Fan et al. 2017). Hence, miR-200b/429 cluster expressions have been proposed as a sensitive and specific marker for diagnostic and prognostic application in cervical cancer (Zeng et al. 2016; KZ et al. 2020). Although miR-200b/429 is a clustered miRNA, none of the previous cervical cancer studies have investigated the diagnostic, prognostic, and functional significance of these miRNA as a single cluster. The present study aimed to test the diagnostic and prognostic significance of miR-200b/429 in cervical cancer using miRNA expression data from TCGA-CESC and gene expression omnibus (GEO). Further, the in silico findings of miR-200b/429 expression were validated in an independent cohort of cervical cancer samples. We have constructed the interaction networks of miR-200b/429 with mRNA, lncRNA, and circRNA. We have predicted the metastatic and prognostic significance of miR-200b/429. Moreover, we have performed functional enrichment, protein–protein interaction network (PPIN) analysis, and hub genes (HG) identification. A network was constructed based on miR-200b/429 and its validated protein-coding targets, and drug–gene interaction was predicted. Thus, our integrated *in silico* analysis has identified the interactome of miR-200b/429 with potential diagnostic and prognostic functions. The novel pathways and interactome may provide insight into a deeper understanding of cervical cancer and an opportunity to translate these findings for improved cervical cancer management.

## Materials and Methods

### Data Source, Collection, and miR-200b/429 Cluster Expression Analysis

**Fig. 1:**
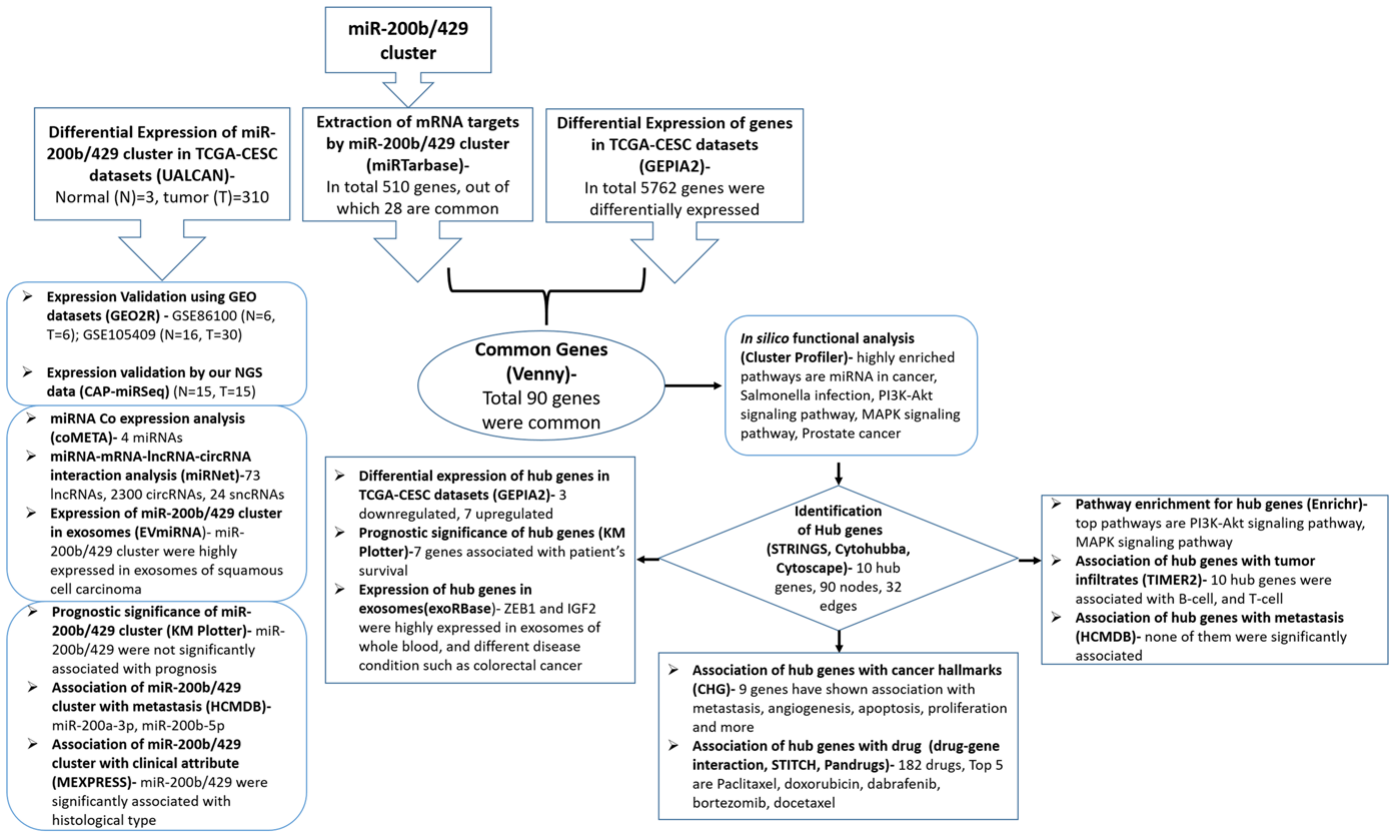
The schematic diagram of in silico analysis of miR-200b/429 cluster, their targets, expression, and associated pathway in CC

The expression of miR-200b, miR-200a, and miR-429 were extracted from TCGA-CESC (normal = 3 samples and tumor = 310 samples) using UALCAN (Chandrashekar et al. 2017) and GEO database (http://www.ncbi.nlm.nih.gov/gds/). We used GEO datasets to strengthen our data as TCGA has fewer normal samples. The GEO datasets analyzed included GSE86100 (6 normal and 6 tumors) (Gao et al. 2016) and GSE105409 (1 normal pool of 16 samples and 1 tumor pool of 30 samples) (Kawai et al. 2018). The differential expression of miR-200b/429 in TCGA-CESC and GEO datasets were analyzed using Transcriptome Alterations in CanCer Omnibus (Chou et al. 2019) and GEO2R tools (https://www.ncbi.nlm.nih.gov/geo/geo2r/), respectively. The associations between members of the miR-200b/429 cluster with clinical parameters were tested by MEXPRESS (Koch et al. 2015). The conservation of this miRNA cluster was predicted using the ECR browser (Ovcharenko et al. 2004). The flowchart for bioinformatic analysis is shown in Fig. 1.

### miR-200b/429 Cluster Target Prediction and Expression Analysis

Co-Expression Meta-Analysis of miRNA target (COMETA) (Gennarino et al. 2012) tool was used to predict the co-expressing miRNAs of the miR-200b/429 cluster. miRTarBase (Huang et al. 2020a, b) was used to predict the miR-200b/429 targets. miRTarBase is a freely available online database consisting of well-annotated and experimentally confirmed miRNA–target interactions. The expression analysis was performed using GEPIA2 (Tang et al. 2019). The genes commonly differentially expressed in multiple datasets were subsequently considered as miR-200b/429 targets. The gene expression in exosomes was performed by exoRBase (Li et al. 2018).

### Functional Enrichment Analysis and Protein–Protein Interaction Network Construction

The in silico functional enrichment (biological functions, gene ontology, and pathway) of miR-200b/429 targets were undertaken using Enrichr. Gene ontology analysis consisted of enrichment for biological process (BP), molecular function (MF), and cellular components (CC) (Xie et al. 2021). A protein–protein interaction network (PPIN) regulated by miR-200b/429 was constructed using STRING V11 (Szklarczyk et al. 2015). PPIN was constructed for differentially expressed targets of miR-200b/429 using known interactions, predicted interactions, text mining, co-expression, and protein homology. The hub genes of the networks were predicted by the cytohubba tool (Chin et al. 2014), and Cytoscape to visualizes networks. Using Cluster Profiler, functional enrichment analysis of genes was performed (Yu et al. 2012). Hub genes-immune association is performed by TIMER2 (Li et al. 2021).

### Systems Biology Analysis/Downstream Analysis

The miR-200b/429 centric lncRNA, circRNA, and sncRNA network was constructed using the miRNet tool (Chang et al. 2020). The parameters used for network construction includes Organism type: Human, ID type: miRbase ID, Tissue type: Cervix, Targets: lncRNA, circRNA, and sncRNA. DIANA-lncbase V3 was used to identify the experimentally validated miRNA–lncRNA interactions in cervical cancer (Karagkouni et al. 2020). The prognostic significance of miR-200b/429 and hub genes was evaluated using the Kaplan–Meier survival curve with the Log-rank method KM plotter tool (Nagy et al. 2021). Using the TACCO tool (Chou et al. 2019), a prognostic model was built using the expression of 10 hub genes for overall survival using the random forest algorithm. HCMDB (Human Cancer Metastasis Database) (Zheng et al. 2018) tool was used to identify the association between miR-200b/429 and hub genes with metastasis.

### Prediction of Drug–Gene Interaction

The differentially expressed targets of miR-200b/429 were used for predicting the druggable genes using Drug-Gene Interaction Database 3.0 (Freshour et al. 2021). The drug–gene interaction network was generated using STITCH (Kuhn et al. 2008) and prioritized using PanDrugs (Piñeiro-Yáñez et al. 2018).

### Validation by Small RNA Sequencing

We had previously performed small RNA sequencing in 15 normal and 15 cervical cancer samples using the P1™ chip using Ion Proton system™ (Shukla et al. 2019). The miRNA alignment and differential expression analysis were undertaken using the CAP-miRSeq pipeline (Sun et al. 2014). The expression of miR-200b/429 was cross-validated using our previously published data (Shukla et al. 2019).

## Results

### miR-200b/429 Cluster is Overexpressed in Cervical Cancer

**Fig. 2:**
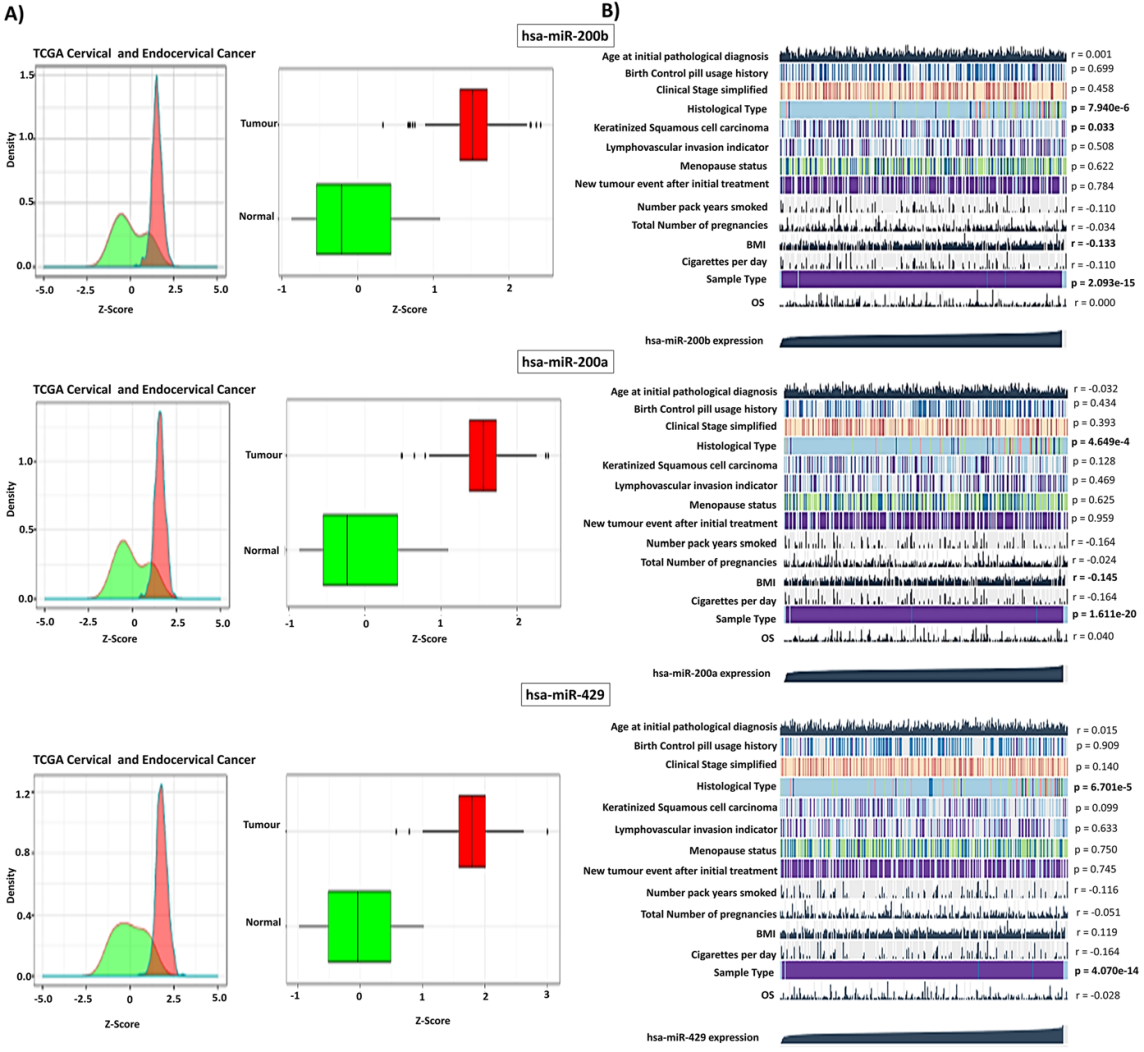
**a** TCGA-CESC datasets have revealed the overexpression of miR-200b, miR-200a, and miR-429 in cervical cancer compared to healthy controls. The analysis was performed using miRNACANCERMAP (Tong et al. 2018), **b** Correlation analysis between clinical attributes and miR-200b/429 expression. miR-200b, miR-200a, and miR-429 expression have shown to be significantly associated with histological type. The analysis was performed using MEXPRESS

**Fig. 3:**
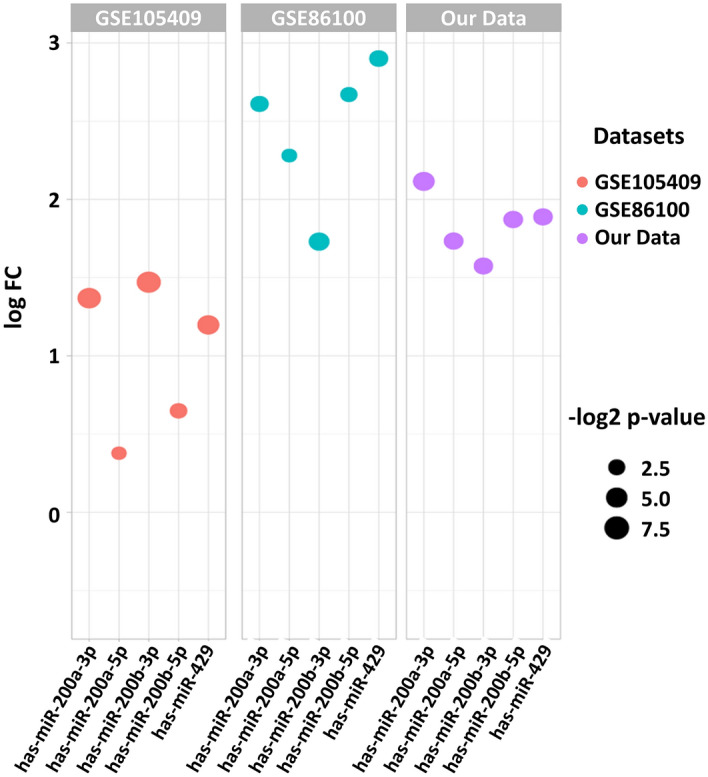
Expression of miR-200b/429 cluster in GEO datasets and our NGS datasets

The miR-200b/429 is located at 1p36 and is a highly conserved miRNA cluster (Supplementary Fig. 1a and 1b). The expression of individual members of the miR-200b/429 cluster was evaluated in TCGA-CESC, GSE86100, and GSE105409, datasets consisting of 24 normal and 346 cervical cancer samples. The miR-200b/429 cluster expression was significantly higher in tumor samples compared to normal samples in TCGA-CESC, GSE86100, and GSE105409 datasets (Figs. 2a, 3). The description for the datasets is shown in Supplementary Table 1 and 2. The MEXPRESS analysis identified that miR-200b/429 expression was significantly correlated with the histological type (*P* < 0.01, Fig. 2b).

### Validation of miR-200b/429 Expression

The miR-200b/429 was cross-validated using our previously published small RNA sequencing data. The expression of miR-200a (1.81-fold), miR-200b (1.53-fold), and miR-429 (1.60-fold) were significantly higher in our small cohort of cervical cancer samples when compared with the normal cervix (Fig. 3). These results further confirm our in silico analysis.

### miR-200b/429 Cluster and Target Network Construction

**Fig. 4:**
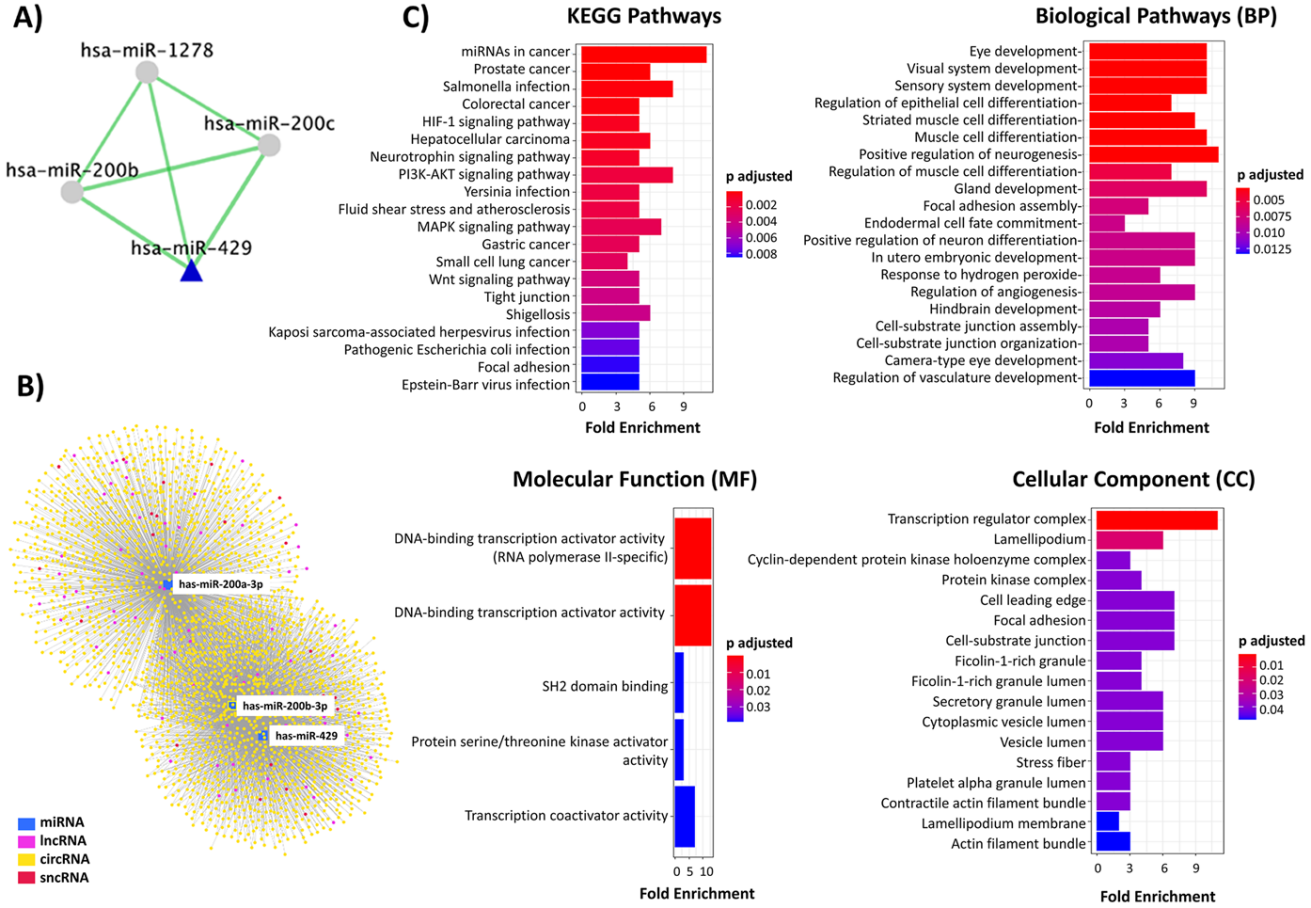
miR-200b/429 cluster in silico analysis. **a** miRNA–miRNA interaction analysis has shown these clusters are interacting with each other along with miR-1278. **b** Construction of cervix specific miR-200b/429 cluster, mRNA, lncRNA, circRNA, and sncRNA network. **c** Functional enrichment analysis of 90 common genes targeted by miR-200b/429 cluster

The miRNA–miRNA co-expression network constructed using the COMETA tool identified miR-200a, miR-200b, miR-429, and miR-1278 as members of the co-expression as the immediate members of the interaction network (Fig. 4a). We next constructed a cervical cancer-specific miR-200b/429—lncRNA–circRNA–sncRNA network by a miRNA-centric network visual analytics platform (miRNet). The miR-200b/429 interacted with 73 lncRNA, 2300 circRNA, and 24 sncRNA consisting of 4333 edges (Fig. 4b). Further, we have identified 31 experimentally validated interactions between miR-200b/429 cluster and lncRNAs in cervical cancer using DIANA LncBase v3. Among 31 interactions, two were common with lncRNAs identified from miRNET analysis, namely, MALAT1 and ATP5F1AP2. The prediction using miRTarBase identified 510 protein-coding genes (miR-200a: 181, miR-200b:210, and miR-429: 119) as targets of miR-200b/429. Interestingly, 28 protein-coding genes were commonly targeted by all three members of this cluster (Supplementary Fig. 1c, Supplementary Table 3 & 4). As per Log_2_FC, Cutoff:1 and q-value Cutoff:0.01 have identified that 5762 genes are differentially expressed between normal and tumor samples in the TCGA-CESC dataset (Supplementary Table 5). The overlapping analysis between 5762 differentially expressed genes (1851 up to and 3911 down) with 510 targets of miR-200b/429 identified 90 differentially expressed genes as targets (Supplementary Fig. 1d & Supplementary Table 6).

### Functional Enrichment Analysis of miR-200b/429

The functional enrichment analysis was undertaken for 90 differentially expressed targets of miR-200b/429. The pathway enrichment was performed by comparing the differentially expressed genes with KEGG annotations. The top ten pathways enriched included microRNAs in cancer, Salmonella infection, PI3K–AKT signaling pathway, MAPK signaling pathway, Prostate cancer, Hepatocellular carcinoma, Shigellosis, Colorectal cancer, HIF-1 signaling pathway, and Neurotrophin signaling pathway. The top ten BP enriched included focal adhesion assembly, gland development, regulation of muscle cell differentiation, positive regulation of neurogenesis, muscle cell differentiation, striated muscle cell differentiation, regulation of epithelial cell differentiation, sensory system development, visual system development, and eye development. The top MF enriched are DNA-binding transcription activator activity, RNA polymerase II-specific DNA-binding transcription activator activity, SH2 domain-binding protein serine/threonine kinase activator activity, and transcription coactivator activity. The top CC enriched included secretory granule lumen, ficolin-1- rich granule lumen, ficolin-1-rich granule, cell–substrate junction, focal adhesion, cell leading edge, protein kinase complex, cyclin-dependent protein kinase holoenzyme complex, lamellipodium, and transcription regulator complex. Besides, the disease gene network construction and disease ontology predicted the differentially expressed genes to participate in cancer (Fig. 4c).

### PPIN of miR-200b/429 Targets

The PPIN of 90 target genes of miR-200b/429 was constructed using STRING v11. The PPIN consisted of 90 nodes and 32 edges with a PPI enrichment p-value of 4.27e-05. The hub genes of the network predicted using cytohubba-identified Enhancer of zeste homolog 2 (EZH2), Fms-related receptor tyrosine kinase 1 (FLT1), Insulin-like growth factor 2 (IGF2), Insulin receptor substrate 1 (IRS1), Jun proto-oncogene, AP-1 transcription factor subunit (JUN), Kinase insert domain receptor (KDR), SRY-box transcription factor 2 (SOX2), MYB proto-oncogene transcription factor (MYB), Zinc finger E-box-binding homeobox 1 (ZEB1), and TIMP metallopeptidase inhibitor 2 (TIMP2) as the top ten hub genes of the target network consisting of 10 nodes, 25 edges with a PPI enrichment p-value of 3.42e-14 (Supplementary Fig. 2). We also performed the 10 hub genes systemic analysis of immune infiltrate across the TCGA-CESC cohort (*n* = 306). Our analysis has shown the association of hub genes with B-cell, T-cell CD4+, and T-cell CD8+ with a p-value < 0.05 (Supplementary Table 7). To determine the gene expression in the same tissue, we used TCGA-CESC datasets to analyze the gene expression. Next, we performed a Pearson correlation analysis of miRNA and mRNA expression from the TCGA datasets using TACCO (Chou et al. 2019) and identified that miR-200b/429 clusters primary targets, ZEB1, IRS1, FLT1 KDR, and TIMP2, as shown in Table 1.

**Table 1:** miRNA:mRNA correlation based on TCGA-CESC datasets

miRNA	Target gene	Pearson’s r	Spearman’s p
hsa-miR-200b-3p	IRS1	− 0.068	− 0.091
hsa-miR-200b-3p	FLT1	− 0.196	− 0.157
hsa-miR-200b-3p	KDR	− 0.167	− 0.126
hsa-miR-200b-3p	ZEB1	− 0.273	− 0.233
hsa-miR-200a-3p	ZEB1	− 0.253	− 0.239
hsa-miR-429	TIMP2	− 0.24	− 0.212
hsa-miR-429	ZEB1	− 0.277	− 0.261

### Expression and Functional Enrichment Analysis of Hub Target Genes of miR-200b/429

**Fig. 5:**
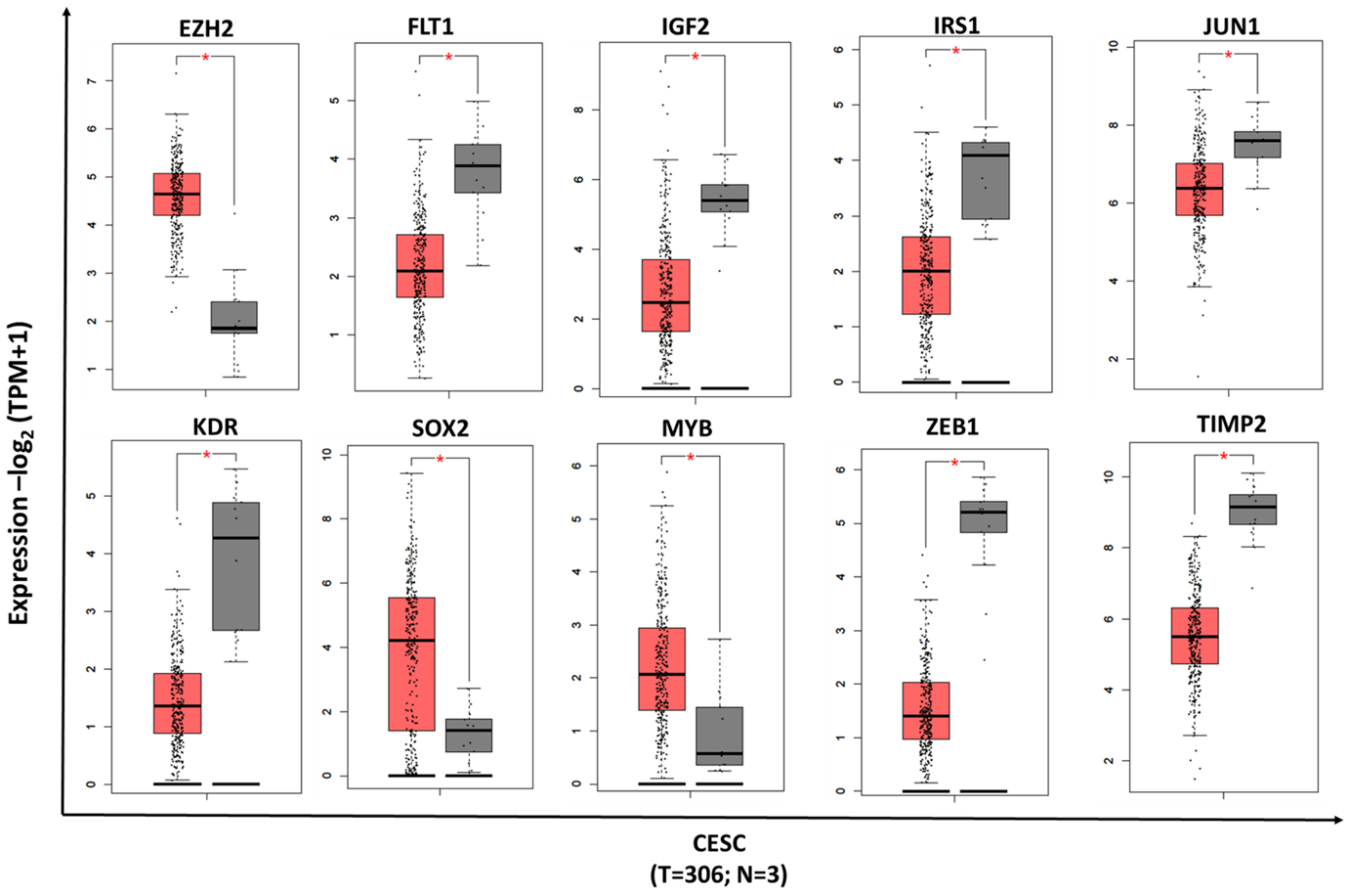
The expression of hub genes in cervical cancer samples compared to normal. *p* < 0.05 were considered significant

The expression of 10 hub genes in normal and cervical cancer samples was tested using the GEPIA2 tool, 306 tumors, and 3 normal samples. EZH2, SOX2, and MYB were significantly upregulated among the hub genes, while FLT1, IGF2, IRS1, JUN, KDR, ZEB1, and TIMP2 were downregulated, respectively (Fig. 5).

The cluster profiler performed the enrichment of hub genes, which included analysis of gene ontology, pathway enrichment, transcription factor mapping, miRNA search, and others. PI3K–AKT signaling pathway and MAPK signaling pathway emerged as major target pathways. (Supplementary Fig. 3).

### Prognostic Significance of miR-200b/429 and Hub Genes

**Fig. 6:**
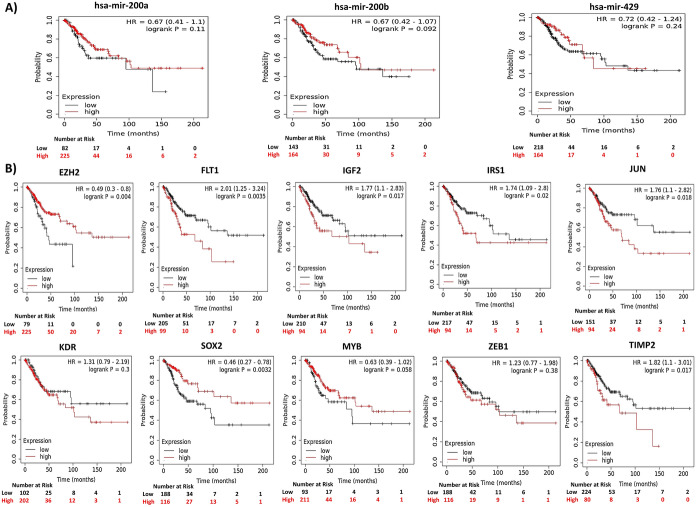
The prognostic significance of miR-200b/429 and hub genes

The KM survival analysis showed that the miR-200b/429 cluster expression did not significantly affect patient survival. We next analyzed the prognostic significance of 10 hub genes in patient survival. Interestingly, the differential expression of 7 genes (EZH2, FLT1, IGF2, IRS1, JUN, SOX2, and TIMP2) was shown to influence patient survival as analyzed by the KM plotter (Fig. 6). Next, we analyzed the role of differential expression of miR-200b/429 and hub gene on cervical cancer metastasis. The expression of miR-200a-3p expression is significantly higher in primary tumors with metastasis than without, with head & neck, and lung being the major metastatic sites. Interestingly, hsa-miR-200b-5p was significantly under-expressed in metastatic cervical cancer samples. However, hsa-miR-200a-5p, hsa-miR-200b-3p, and hsa-miR-429 expressions were not differentially expressed between primary tumors with metastasis compared to those without metastasis. None of the hub genes were significantly associated with metastasis (Supplementary Fig. 4).

### miR-200b/429-Hub and Cancer Hallmarks

We next investigated the potential of ten hub genes in the induction of cancer hallmarks using CHG online tool. Among the ten hub genes, only data were available for 9 genes. The CHG analysis showed that abnormal expression of hub genes (EZH2, FLT1, IGF2, IRS1, JUN, KDR, SOX2, MYB, and ZEB1) might facilitate participating in pathways leading to the promotion of growth, sustained proliferation, resistance to apoptosis, induction of angiogenesis, activation of invasion, and metastasis, enabling replicative immortality, evading immune destruction, and tumor-promoting inflammation (Supplementary Table 8).

### Drug–Gene Interaction Analysis

The potentially druggable genes of miR-200b/429 targets were predicted using the DGIdb database. Screening using DGIdb predicted 27 genes and 182 potential drugs. Furthermore, the interaction network of 27 genes using STITCH identified 34 nodes and 41 edges with a PPI enrichment p-value of 2.02e-05. Paclitaxel, doxorubicin, dabrafenib, bortezomib, docetaxel, ABT-199, eribulin, vorinostat, etoposide, and mitoxantrone as the top 10 best candidate drugs emerged out of Pandrug analysis (Supplementary tables 9 and 10).

## Discussion

Despite the progress and improvement in diagnosis and prognosis, cervical cancer is one of the key public health problems in many developing and under-developed countries (Hull1 et al. 2020). Infection with HPV and genetic and epigenetic changes in both host and HPV play a critical role in the development and progression of cervical cancer (Senapati et al. 2016). Pap screening and HPV vaccines have helped to reduce the cervical cancer burden globally. However, several countries still have high incidence and mortality due to cervical cancer (Arbyn et al. 2020). The lack of effective screening programs, socioeconomic status, early cancer detection, the tendency to metastasis, and therapy resistance are some critical factors contributing to the high incidence and mortality of cervical cancer (Mishra et al. 2011). Thus, understanding this disease at the molecular level is critical for improved diagnosis, prognosis, and finding alternative drugs and targets for effectively managing cervical cancer. Recent progress in systems biology, genome-wide studies, and big data in public domains provide a platform to identify the key molecular changes that occur during tumorigenesis and provide an opportunity to understand the genes and pathways responsible for carcinogenesis and theragnostic applications. miRNAs are small non-coding RNAs critical for regulating the expression of protein-coding genes. We and others have shown that abnormal miRNA expression occurs during cervical carcinogenesis, and profiling can be used as a potential diagnostic and prognostic indicator (Pereira et al. 2010). By analyzing the publicly available miRNA datasets in TCGA and GEO, we aimed to identify the prognostic potential of the miR-200b/429 cluster in cervical cancer. Additionally, we have predicted the target genes, hub genes, pathways, interaction, and biological function using in silico approach. Our study demonstrated that miR-200b/429 overexpression might promote cervical cancer via activation of PI3K–AKT and MAPK signaling pathways. Besides, the targets of miR-200b/429 have prognostic value in cervical cancer. Furthermore, either the deregulated expression of miR-200b/429 or its target genes can predict metastasis in cervical cancer. Our study has identified several novel genes as targets of FDA-approved drugs.

Through integrated bioinformatic analysis using 4 miRNA expression datasets, we first showed that miR-200b/429 is overexpressed in cervical cancer compared to normal control samples and may act as an oncogene and promoter of cervical cancer. Furthermore, our study showed that measuring miR-200b/429 could be helpful for the diagnosis and histological classification of cervical cancer. The three miRNAs of the cluster were differentially expressed in primary tumors with metastasis compared to those without metastasis. Thus, measuring the miR-200b/429 expression could be useful as a predictor of metastasis in cervical cancer. However, the miR-200b/429 cluster expression did not correlate with patient survival.

The hsa-miR-200b, the first member of the cluster (miR-200b/429), has been shown to promote cervical cancer cell proliferation and metastasis by inhibiting Forkhead box G1 (FOXG1) (Zeng et al. 2016). miR-200b was also shown to be hypomethylated and target Homeodomain-interacting protein kinase 3 (HIPK3) in cervical cancer (Varghese et al. 2018). Downregulation of miR-200b increased cell apoptosis and inhibited tumor growsth in vivo (Zeng et al. 2016). Hsa-miR-200a was upregulated in HPV-positive cervical tissues (Mandal et al. 2019; Vojtechova et al. 2016).

In contrast to its oncogenic function, other studies have shown its tumor-suppressive roles. For example, a study by Cheng et al. 2016 has shown miR-200b to suppress cell invasion and metastasis via inhibiting epithelial to mesenchymal transition (EMT) in cervical cancer (Cheng et al. 2016). Moreover, a study by He et al. 2020 has shown that overexpression of hsa-miR-200b increases apoptosis (He et al. 2020).

Hsa-miR-200a has been shown to downregulate metastatic genes such as ZEB1, and ZEB2, which is suggested to suppress the migration of cervical cancer cells (Hu et al. 2010). Similarly, hsa-miR-429 targets the inhibitor of nuclear factor Kappa B kinase subunit beta (IKKβ) and regulates NF-kB, suggesting its tumor-suppressive function (Fan et al. 2017). Hence, this cluster was shown to play an essential role in the progression of cervical cancer and may play an important role in the future as a biomarker. miR-200b/429 cluster target gene prediction was carried out for a deeper understanding of its function and led to the identification of 312 target genes. Further screening of 312 target genes in the TCGA-CESC dataset identified 90 target genes as differentially expressed and was subsequently used for downstream analysis. A PPIN of 90 genes identified FLT1, IGF2, IRS1, JUN, KDR, ZEB1, TIMP2 (downregulated), and EZH2, SOX2, and MYB (upregulated) in cervical cancer samples when compared with normal samples. Among the 10 hub genes, the expression of EZH2, FLT1, IGF2, IRS1, JUN, SOX2, and TIMP2 individually correlated with cervical cancer patient survival. Very interestingly, an overall survival model based on 10 hub genes using a random forest approach can be used to identify the high-risk and low-risk categories with a sensitivity and specificity of 0.92 and 0.93, respectively (Supplementary Fig. 5). Besides, the expression of 7 genes (EZH2, FLT1, IRS1, KDR2, MYB2, JUN, and TIMP2) was significantly different in metastatic tissue when compared with the primary tumor. Among the hub genes, except for TIMP2, the other 9 genes were associated with the induction of various cancer hallmarks. These data indicate that miR-200b/429 and the associated hub gene network may facilitate the acquisition of multiple cancer hallmarks to promote cervical cancer. We anticipate that measuring miR-200b/429 and hub gene expression may be useful for diagnosis, prognosis, histological evaluation, and prediction of metastasis in cervical cancer.

We next performed a functional enrichment analysis of miR-200b/429 for a deeper understanding of its role in cervical cancer. We showed that miR-200b/429 could regulate key cell signaling pathways, specifically the PI3K−AKT and MAPK signaling pathways. Activation of PI3K−AKT and MAPK signaling pathways are often activated in cervical cancer, and its role is well established in cervical cancer (Zhang et al. 2015). The enriched BP, MF, and CC terms are often associated with cancer.

Therapy resistance is the major factor associated with clinical outcomes and patient survival. Many previous studies have shown that both intrinsic and acquired resistance to conventional chemotherapeutic agents is one of the critical problems in cancer treatment and patient survival (Vasan et al. 2019). Hence, it is essential to understand the genes and pathways leading to therapy resistance for better management of cancer patients. Towards this, we have performed the drug–gene interaction analysis and identified 27 druggable genes and 182 potential drugs. Furthermore, prioritization identified 10 FDA-approved drugs as the best candidate for targeting miR-200b/429 and associated networks. However, more detailed clinical studies are required before further conclusions are drawn. Nevertheless, our study has identified genes, pathways, and new targets amenable for future studies. Thus, the new drugs predicted in our study may find new clinical use in cervical cancer. miRNAs have now also been shown to interact with other non-coding RNAs such as lncRNAs and circRNAs. For this reason, it is essential to understand the complex connections of the miR-200b/429 cluster in cervical cancer. With this, we have predicted miRNA interactions with lncRNAs and circRNAs. LncRNAs, which modulate gene expression, are now emerged as a new class of functional regulatory elements and have been associated with diverse human diseases. lncRNAs have been shown to contain miRNA-binding sites and exert their functions through titrating other miRNAs (Paraskevopoulou and Hatzigeorgiou 2016). These lncRNAs have been shown to act as sponges for miRNAs to reduce their activity. For example, lncRNA TPT1-AS1 sponges miR-324-5p and promotes cell growth and metastasis in cervical cancer (Jiang et al. 2018). Therefore, we have predicted miR-200b/429 cluster-binding sites on lncRNA and found XIST and MALAT1 to be common targets. Studies have shown that lncRNA XIST promotes cervical cancer progression by upregulating FUS via competitively binding to miR-200a (Zhu et al. 2018). lncRNA MALAT1 promotes cervical cancer through sponging miR-429 (Shen et al. 2019).

Similarly, circRNA can regulate gene expression by sponging miRNA. For example, circRNA cSMARCA5 acts as a sponge for miR-432 and regulates cervical cancer progression (Huang et al. 2020b). Towards this, we have predicted miRNA-binding sites on circRNA and identified six common circRNAs (MORC3_hsa-circRNA5148, CDK17_hsa-circRNA10073, TP53INP1_hsa-circRNA15552, USP9X_hsa-circRNA7984, XPO4_hsa-circRNA10219, and SEC23A_hsa-circRNA10433). The interaction of these circRNAs with miR-200b/429 needs further experimentation and validation. There are also reports showing the destruction of miRNA by lncRNA, which ultimately accumulated the circRNA. For exp, lncRNA Cyrano directs the destruction of miR-7 and accumulated circRNA Cdr1as in the cytoplasm of neurons (Kleaveland et al. 2018). In this study, we focused on miRNA–lncRNA and miRNA–circRNA interaction analysis based on miRNA-binding sites on lncRNA and circRNA. Further, the relationship between miRNA–mRNA–lncRNA–circRNA needs to be established to explore the crosstalk between non-coding RNAs. The understanding of miRNA's complex networks will be the starting point to unraveling their role in tumorigenesis and disease progression. Given the significance of the mRNAs, lncRNAs, and circRNAs discovered in our work and other prior findings, more research into the involvement of these coding and non-coding RNAs in the pathophysiology of cervical cancer could be attempted. Our study made use of genome-wide data from public sources as well as freely available web tools; validation through experimental approach must be assessed to improve the miRNA-based therapy in the clinic.

## Conclusion

Using the available miRNA and transcriptomics datasets, we have identified the prognostic value, biological function, and signaling pathways of miR-200b/429 and their target hub genes in cervical cancer. Altogether, we have identified the miR-200b/429 and associated gene regulatory networks that may offer a promising candidate for diagnosis, prognosis, and therapeutic application in cervical cancer. Our study did have certain limitations. Firstly, all the analysis was carried out using freely available online tools that analyzed the TCGA-CESC dataset. The TCGA-CESC dataset contained only three normal samples, is another limitation of the study. It was challenging to consider demographic aspects such as diverse age groups, ethnicities, geographical locations, tumor stage and classification for all the patients while assessing the DEGs due to the complexity of the datasets in our study. Secondly, all the analyses performed were in silico analysis that requires further validation. Therefore, further research is needed to determine their mechanistic basis. MiRNAs exert different functions in different tissue. Furthermore, miRNAs target genes show differential expression in different tissue types. Thus it is important to perform a correlation analysis between miRNA and its target gene expression from the same tissue. In our study, we have not used the miRNA–target gene expression from the same tissue, which is one of the study's limitations.

Regarding its function in cervical cancer, ZEB1, which has been identified as a common gene controlled by the miR-200b/429 cluster, requires more investigation. The bioinformatics analysis used in this work to examine the overall survival and expression levels of the ten hub genes may aid in developing a biomarker panel for this patient population. We still require larger, prospective studies to validate these hub genes to identify their sensitivity and specificity, particularly in cervical cancer. We have also discovered that hub genes are present in exosomes from healthy individuals, whole blood, and various disease conditions like colorectal cancer and pancreatic adenocarcinoma. However, to confirm the existence of a minimally invasive cervical cancer-specific biomarker, we must further validate the expression of these hub genes in cervical cancer serum or plasma samples.

### Supplementary Information

Below is the link to the electronic supplementary material.

Supplementary file1 (TIF 3123 KB)—Information about miR-200b/429 cluster. a) Genomic organization of miR-200b/429 cluster. b) Conservation analysis for miR-200b/429, c) Venny analysis of common genes targeted by miR-200b/429 cluster, and d) common genes targeted by miR-200b/429 cluster and significant differential expressed genes in TCGA-CESC datasets

Supplementary file2 (TIF 8037 KB)—a)The PPIN of miR-200b/429 cluster. b) Identification of hub genes by protein-protein interaction analysis

Supplementary file3 (TIF 9452 KB)—The pathway enrichment analysis for 10 hub genes

Supplementary file4 (TIF 8342 KB)—a and b) Association of miR-200b/429 and hub genes with cervical cancer metastasis

Supplementary file5 (TIF 3252 KB)—Overall survival model based on 10 hub genes

Supplementary file6 (XLSX 11 KB)—Details of the datasets used in the study

Supplementary file7 (XLSX 35 KB)—TCGA_CESC- Sociodemographic and clinical information

Supplementary file8 (XLSX 15 KB)—List of genes targeted by miR-200b/429 cluster

Supplementary file9 (XLSX 9 KB)—Common genes targeted by miR-200b/429 cluster

Supplementary file10 (XLSX 347 KB)—List of differentially expressed genes in TCGA_CESC

Supplementary file11 (XLSX 10 KB)—Common Genes targeted by mir-200b/429 and differential expressed in TCGA_CESC datasets

Supplementary file12 (XLSX 17 KB)—Association of hub genes with B-cell, T-cell CD4+, and T-cell CD8+

Supplementary file13 (XLSX 11 KB)—Association of hub genes with cancer hallmarks

Supplementary file14 (XLSX 21 KB)—List of Druggable genes

Supplementary file15 (XLSX 17 KB)—Gene-drug interactions

## Data Availability

Not Applicable.
