# Supplementary figures and images for: Bioinformatic Analysis of miR-200b/429 and Hub Gene Network in Cervical Cancer
Source: Biochem Genet. 2023 Mar 7;61(5):1898–916. doi: 10.1007/s10528-023-10356-2 (PMC10517900; doi:10.1007/s10528-023-10356-2)

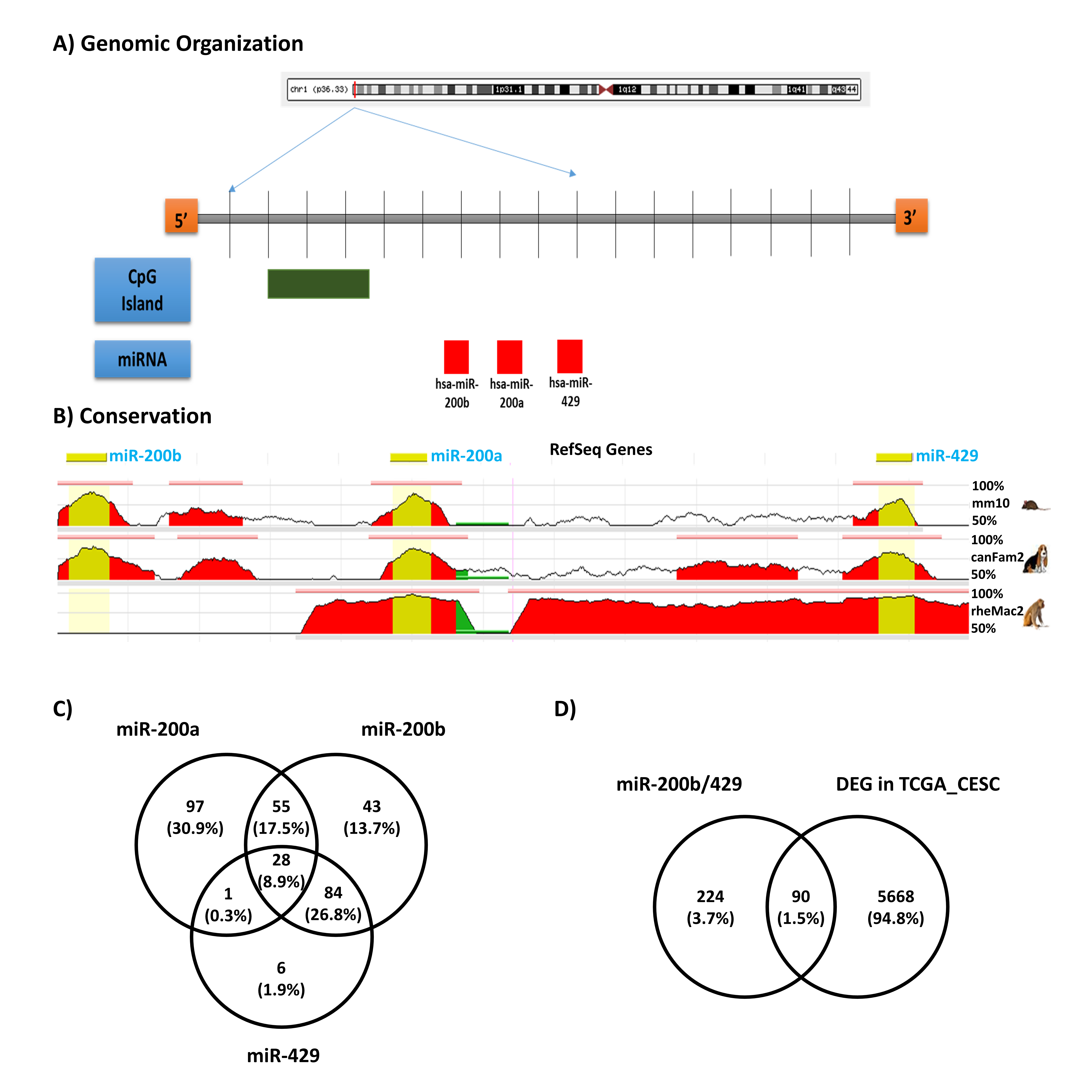

Supplement: Supplementary file 1 — Supplementary file1 (TIF 3123 KB)—Information about miR-200b/429 cluster. a) Genomic organization of miR-200b/429 cluster. b) Conservation analysis for miR-200b/429, c) Venny analysis of common genes targeted by miR-200b/429 cluster, and d) common genes targeted by miR-200b/429 cluster and significant differential expressed genes in TCGA-CESC datasets [file 10528_2023_10356_MOESM1_ESM.tif]

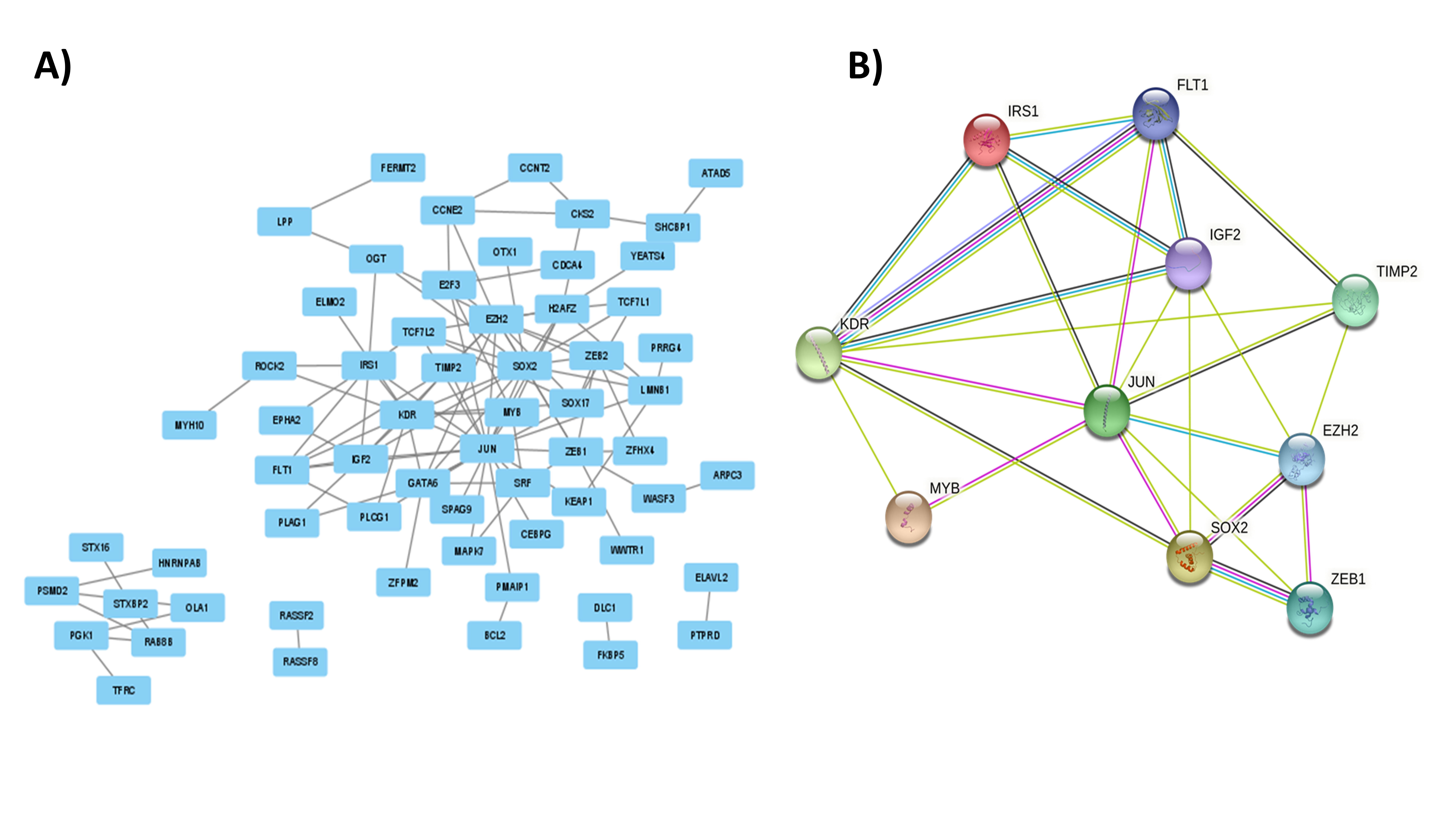

Supplement: Supplementary file 2 — Supplementary file2 (TIF 8037 KB)—a)The PPIN of miR-200b/429 cluster. b) Identification of hub genes by protein-protein interaction analysis [file 10528_2023_10356_MOESM2_ESM.tif]

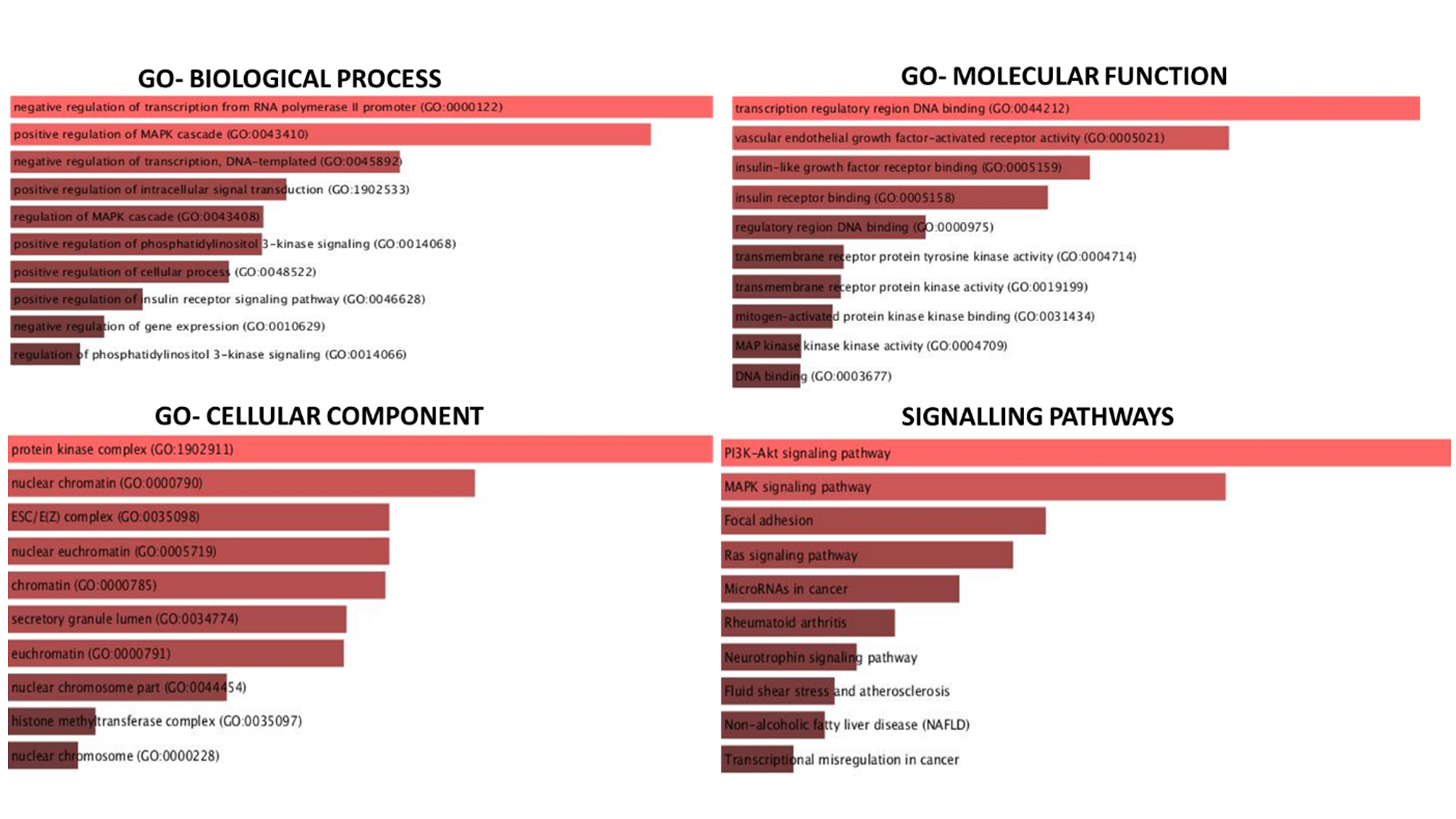

Supplement: Supplementary file 3 — Supplementary file3 (TIF 9452 KB)—The pathway enrichment analysis for 10 hub genes [file 10528_2023_10356_MOESM3_ESM.tif]

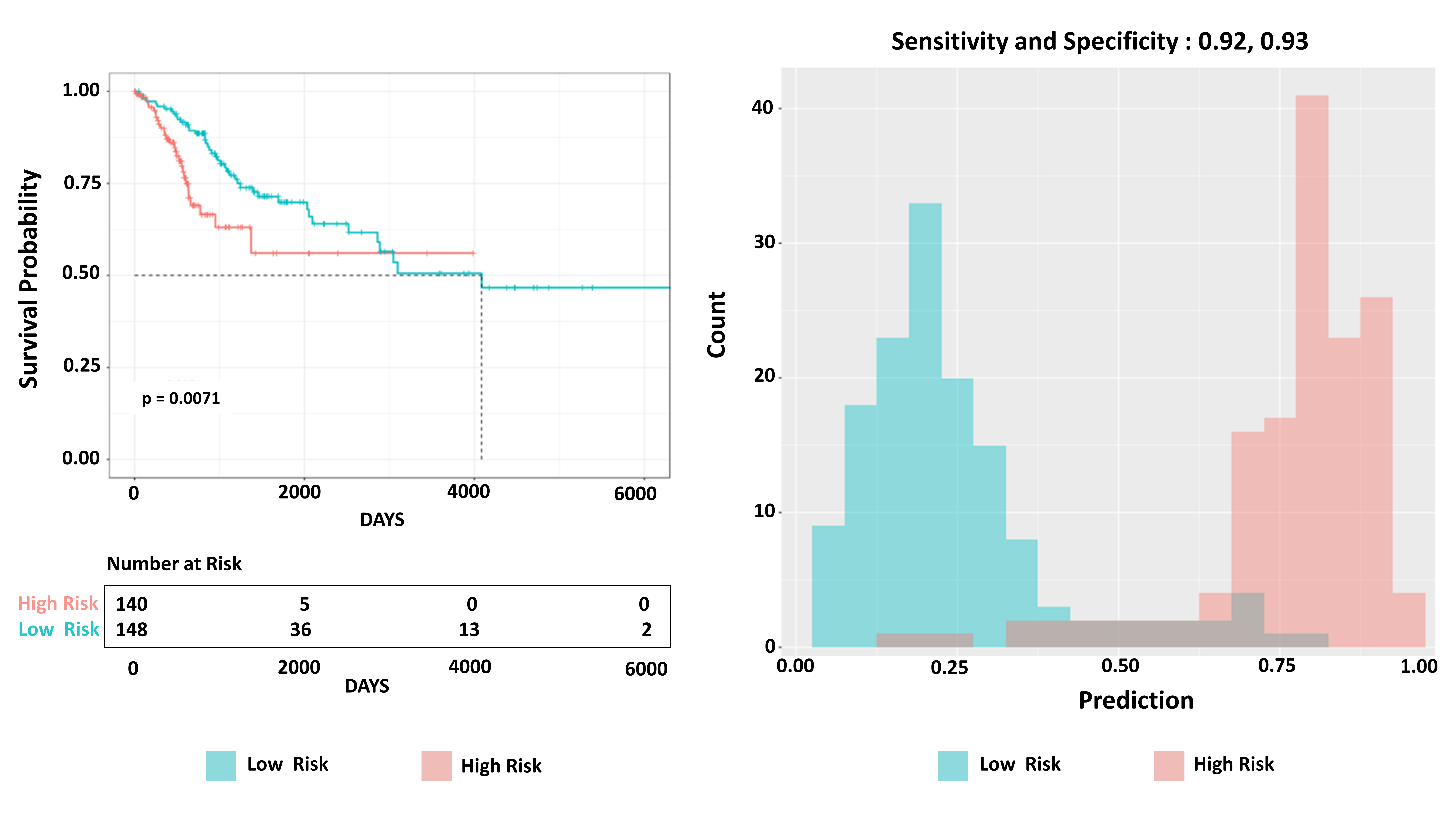

Supplement: Supplementary file 5 — Supplementary file5 (TIF 3252 KB)—Overall survival model based on 10 hub genes [file 10528_2023_10356_MOESM5_ESM.tif]
